# The role of ketamine and its enantiomer in managing depression and pain in cancer patients: A narrative review

**DOI:** 10.1016/j.jatmed.2024.10.005

**Published:** 2024-11-29

**Authors:** Michael S. Bodnar, Sierra Barber, Heather S.L. Jim, Jeffery Huang

**Affiliations:** a University of South Florida Morsani College of Medicine, Tampa, FL 34433, USA; b Department of Health Outcomes and Behavior, Moffitt Cancer Center, Tampa, FL 34433, USA; c Department of Anesthesiology, Moffitt Cancer Center, Tampa, FL 34433, USA

**Keywords:** Ketamine, Cancer, Depression, Pain Management, Anesthesia

## Abstract

Depression and pain are common comorbidities in cancer patients, and ketamine, a dissociative anesthetic, has shown potential in managing both. This review summarizes current literature on ketamine and its enantiomer, esketamine, in managing depression and pain in the oncologic population. Studies indicate that sub-anesthetic doses of intravenous ketamine and esketamine can alleviate postoperative depressive symptoms in cancer patients with a tolerable safety profile. Research into non-intravenous routes for depression management in the oncologic population remains limited. Ketamine has also proven effective in managing acute postoperative pain, particularly through intravenous administration. While alternative administration routes, such as local infiltration and intramuscular methods, show mixed results, they may provide viable options for patients averse to intravenous (IV). However, the effectiveness of ketamine for chronic cancer pain remains inconsistent. Overall, ketamine offers a promising approach for managing depression and pain in oncologic patients.

## Introduction

Initially recognized for its anesthetic properties, ketamine’s unique pharmacodynamic characteristics have garnered significant interest in indications beyond the operating room. Notably, ketamine has demonstrated remarkable promise in the management of both depression and pain management, providing a new tool to help manage two historically difficult medical challenges.^1^, ^2^ Worldwide, an estimated 20 million individuals are diagnosed with cancer each year, a number that is expected to rise significantly over the next decade.^3^ Patients diagnosed with cancer are at an increased risk for comorbid depression and chronic pain, making them uniquely positioned to benefit from advances in these domains.^4^ This review presents an overview of current research on ketamine’s effectiveness and limitations in managing depression and acute/chronic pain in oncologic patients.

## Depression

### Anti-depressant actions of ketamine

Ketamine primary functions an *N*-methyl-D-aspartate (NMDA) antagonist through at least two distinct mechanisms. It binds non-competitively to open NMDA channels, occluding the channel and reducing mean opening time. It also binds non-competitively to an allosteric site, decreasing the frequency of channel opening.^5^, ^6^ Secondary to NMDA, ketamine has been shown to affect a multitude of other sites, including L and T -type voltage gated calcium channels, voltage gated sodium and potassium channels, neuronal pacemaker channels, and α-amino-3-hydroxy-5-methyl-4-isoxazolepropionic acid (AMPA) receptors. It modifies opioid and gamma-aminobutyric acid (GABAa) transmission and also modulates several downstream signaling processes, including mammalian target of rapamycin (mTOR) and protein kinase C (PKC).^6^ Ketamine also increases brain derived neurotrophic factor (BDNF) production.^6^, ^7^

Enhancement of excitatory neurotransmission is a common downstream effect that is essential for the effectiveness of both classical and rapid acting antidepressants. It is theorized that ketamine produces its antidepressant effects through a mechanism secondary to NMDA modulation. This is supported by studies demonstrating NMDA receptor-independent antidepressant effects of ketamine metabolites, and a lack of research supporting anti-depressive benefits from other NMDA antagonists, including memantine and nitrous oxide.^8^, ^9^ Ketamine has been shown to decrease default mode network activation (a state of wakeful restlessness overactivated in depressed individuals) and induce long term changes in neuronal connectivity patterns.^10^, ^11^ Ketamine’s antidepressant effects are rapid and can persist for several weeks following administration.^2^ While the antidepressant effects of ketamine are well reported,^12^ the efficacy of different formulations, including intravenous, intranasal, and oral administrations, and their effect on differing populations, including oncologic patients, remains unclear.

### Cancer and depression

Individuals diagnosed with cancer experience a greater risk of depression and suicidal ideation relative to the general population.^4^, ^13^ Comorbid depression can affect many aspects of patient care, leading to increased risk of mortality, reduced adherence to oncological treatments, higher incidences of postoperative complications and pain, extended hospitalizations, and decreased quality of life.^14^, ^15^, ^16^, ^17^, ^18^ The current mainstays of depression management in oncologic patients are a combination of psychotropic medications, including SSRIs, SNRIs, atypical antidepressants such as mirtazapine, and psychotherapeutic interventions.^19^ Although often efficacious, both psychotropic and psychotherapeutic interventions typically require several weeks to show clinical improvement, and many patients still may not experience benefits from these therapies.^19^ Ketamine’s rapid antidepressant effects and reported efficacy in treatment-resistant depression may provide a powerful addition to the range of existing therapies, especially in patients at the end of life, for whom every moment is crucial.

## Ketamine and depression in cancer patients

### Intravenous ketamine

Several studies have assessed the efficacy and safety of intravenous (IV) ketamine or esketamine (the dextrorotary enantiomer of ketamine, known by esketamine) for depression management in patients with cancer. We conducted a literature search in MEDLINE® (via PubMed®), EMBASE, and Scopus from 1 January 1982–20 October 2022.^20^ Out of the 1453 articles identified, only 5 articles (Table 1)^21^, ^22^, ^23^, ^24^, ^25^ were randomized controlled trials (RCT). All studies that met inclusion criteria were conducted in inpatient settings. These studies included various types of cancer, including breast, cervical, and colorectal cancer. Outcomes included the Hamilton Depression Rating Scale (HAM-D), suicidal ideation using the Beck Scale for Suicidal Ideation (BSS), the suicidal section of the Montgomery-Asberg Depression Rating Scale – Suicidal Ideation (MADRS-SI), Montgomery-Asberg Depression Rating Scale (MADRS), and the Hospital Anxiety and Depression Scale (HADS).

**Table 1:** Summary of randomized controlled trials assessing ketamine for depression in cancer patients.

**Citation**	**Dosage and Route of Administration**	**Timing of Administration**	**Primary Outcome**	**Secondary Outcomes**	**Conclusion**	**Duration of Effect**
Fan et al. (2007)^21^	IV Racemic ketamine (0.5 mg/kg)	During inpatient rehabilitation following newly diagnosed cancer	Statistically significant decrease in suicidal ideation at 1- and 3-days post administration	Significant decrease in depression severity (MADRS-SI) 1 day post administration. No adverse events	Racemic (0.5 mg/kg) ketamine reduced suicidal ideation in newly diagnosed cancer patents	3 days
Ren et al. (2022)^22^	IV Racemic ketamine (0.1 mg/kg, 0.2 mg/kg, 0.3 mg/kg)	5 min prior to surgery	Statistically significant decrease in anxiety and depressive scores up to 3 days in the 0.3 mg/kg dose group	Lower serum levels of IL-6, IL-8, and TNF-α for 0.3 mg/kg dose. No adverse events	Racemic ketamine (0.3 mg/kg) reduced anxiety and depression when administered immediately before surgery	3 days
Lui et al. (2021)^23^	IV Racemic ketamine (0.125 mg/kg) or s-ketamine (0.125 mg/kg)	During analgesia induction phase of surgery	Significant reduction in depressive scores at 3 days, 1 week, 1-month post-surgery; effect not persistent at 3 months	Elevated serum levels of BDNF and 5-HT at 3 days, 1 week, 1-month post-surgery. No adverse events	esketamine is more effective than racemic ketamine in reducing post-operative depression; with effects lasting up to once month	1 month
Wang et al. (2020)^24^	IV Racemic ketamine (0.5 mg/kg) or esketamine (0.5 mg/kg, 0.25 mg/kg)	1 h after start of anesthesia	Decreased depression scores for 3 days post-surgery for each treatment group: effect greater in esketamine (0.5)	Increased BDNF and 5-HT levels up to 3 days post-surgery for 0.5 mg/kg S-ketamine group. No adverse events	esketamine (0.5 mg/kg) reduced depressive symptoms when administered intraoperatively. No significant benefit for 0.5 mg/kg racemic or 0.25 mg/kg esketamine groups.	3 days
Xu et al. (2017)^25^	IV Ketamine hydrochloride (0.5 mg/kg)	1 h after start of anesthesia	Decreased depression scores for 3 days post-surgery	No adverse events reported	Sub-anesthesia ketamine (0.5 mg/kg) reduced post-op depressive symptoms when administered intra-operatively	3 days

Fan et al. published a RCT examining subanesthetic doses of IV racemic ketamine and its effect on suicidal ideation. They assessed suicidal ideation one-, three-, and seven-days post administration in 37 patients with either lung, gastric, bone, or pancreatic cancer during inpatient rehabilitation following newly diagnosed cancer. Ketamine was dosed at 0.5 mg/kg over 40 min in the experimental group, and the control group was given 0.05 mg/kg midazolam over an equivalent period. They found a statistically significant decrease in suicidal ideation, measured using the BSS and MADRS-SI, at one- and three-days post ketamine administration. Depression severity, measured as a secondary outcome via the MADRS, was decreased one day post ketamine infusion. Results did not maintain statistical significance at seven days post administration, and no adverse outcomes were reported.^21^

These findings are consistent with other studies assessing ketamine’s impact on depressive and anxiety symptoms in cancer patients, including the work by Ren et al. Researchers which assessed anxiety and depression following pre-operative administration of ketamine in patients with colorectal cancer.^22^ In their study, 104 patients with colorectal cancer were administered IV racemic ketamine, dosed at 0.1 mg/kg, 0.2 mg/kg, or 0.3 mg/kg, or a saline placebo five min prior to the start of surgery. The primary outcome of the study was anxiety and depressions ratings, assessed via the HADS at one, two-, and three-days post-surgery. Secondary outcomes included serum levels of inflammatory biomarkers IL-6, IL-8, and TNF-a. The study found a statistically significant decrease in depression and anxiety scores at each timepoint in the group dosed at 0.3 mg/kg when compared to placebo. No difference was found between the groups receiving 0.1 mg/kg or 0.2 mg/kg when compared to placebo. In the group that received 0.3 mg/kg ketamine, serum levels of IL-6, IL-8, and TNF-α were lower than those in the control group. The study found no differences in recovery times, cough, agitation, or delirium, with no reported adverse events. The authors concluded that a single dose of 0.3 mg/kg ketamine five min before surgery can improve postoperative anxiety and depression for at least three days post-procedure in colorectal cancer patients.^22^

Liu et al. examined ketamine’s antidepressant effects when administered during anesthesia induction phase of surgery, showing a longer-lasting impact compared to previous studies.^23^ In this RCT, 303 breast cancer patients with mild to moderate depression received either 0.125 mg/kg racemic ketamine, 0.125 mg/kg esketamine, or saline. Depression, measured using HAM-D, was assessed at three days, one week, one month, and three months post-surgery. Both ketamine groups had lower depression scores than the control group at three days, one week, and one month, but not at three months. Esketamine was more effective than racemic ketamine. Serum BDNF and 5-hydroxytryptamine (5-HT) was elevated in both treatment groups. No adverse events or surgical complications were reported. The authors concluded that ketamine reduced post-op depression for up to one month, with esketamine showing greater effectiveness.^23^

Additional studies have focused on intra-operative administration. Wang et al. conducted an RCT assessing the intra-operative administration of ketamine in 417 patients with cervical carcinoma during laparoscopic hysterectomies.^24^ Patients were randomized to receive 0.5 mg/kg racemic ketamine, 0.5 mg/kg esketamine, 0.25 mg/kg esketamine, or placebo. Doses were administered one hour after the start of anesthesia. Depression was assessed via the HAM-D 1-, 2-, 3-, 5- and 7-days post procedure. Serum BDNF and 5-HT levels were measured as secondary outcomes. They found that HAM-D scores decreased, and BDNF/5-HT increased in each treatment group up to 3 days after surgery, with the esketamine group experiencing the largest change from baseline. The effect did not persist on days 5 or 7 in any of the treatment groups. Again, no adverse outcomes were observed.^24^

Finally, a RCT from Xu et al. of 50 patients examined the effect of intra-operative ketamine administration on breast cancer patients with depression.^25^ They observed that a single intra-operative dose of 0.5 mg/kg of ketamine hydrochloride administered one hour post anesthesia induction was associated with decreased HAM-D scores at one- and three-days post-surgery when compared to placebo, but the effect did not persist at 7 days post-surgery. They also report no adverse outcomes.^25^ Still, other research in the non-oncologic population have not demonstrated significant changes in depression when ketamine is administered intraoperatively, suggesting more research is expected to fully delineate the effect intra-operative ketamine has on post-operative depression.^26^ Table 1 summarizes multiple randomized controlled trials that assessed ketamine for depression in cancer patients.

### Intranasal esketamine

In 2019, the FDA approved intranasal esketamine for the management of treatment-resistant depression or major depressive disorder (MDD) with acute suicidal ideation.^27^ Surprisingly, while multiple studies have assessed IV ketamine in the oncologic population, alternative routes of administration, including nasal and oral, have not been thoroughly explored.^20^, ^28^ To date, only a single open-label phase II clinical trial (Intranasal Ketamine for Depression in Patients with Cancer Receiving Palliative Care, INKeD-PC Study) has assessed intranasal ketamine’s effectiveness in managing moderate to severe MDD in oncologic patients.^29^ Their study was conducted on palliative care patients with advanced-stage cancer in the inpatient setting, and patients were given three flexible doses of intranasal ketamine ranging from 50–150 mg over a one-week period. Although the study showed rapid reduction depressive symptoms from baseline (baseline mean MADRS of 31, day 8 MADRS of 11, p < 0.001), the study consisted of just twenty volunteers and lacked a control group.^29^ Larger, more rigorous randomized controlled trials are needed to fully support the safety and efficacy of intranasal ketamine to treat depression in this patient population.

### Oral ketamine

In addition to intranasal ketamine, oral ketamine-assisted therapy is offered as a therapy for treatment-resistant depression. Although oral ketamine is not FDA-approved for treating depression, several companies have begun offering these treatments via mail for at-home use, and providers should be aware of their growing popularity. To date, there are no RCTs assessing the use of oral ketamine for managing treatment-resistant depression or major depressive disorder in the oncologic population. Research on the efficacy of oral ketamine in depression management in non-oncologic patients has shown short-term benefit and a tolerable safety profile. However, most of the research has been of low quality, with a small number of total studies, small sample sizes, short length of follow-up, and heterogeneity of treatment protocols.^30^, ^31^, ^32^, ^33^ Oral ketamine has low bioavailability (15–25 %) and undergoes extensive first pass metabolism, a process largely bypassed with IV and nasal administration.^34^, ^35^ One RCT of 214 patients used oral ketamine to treat patients with cancer-related neuropathic pain.^36^ Ketamine dosage was titrated to a tolerable dosage over two weeks, ranging between 40 mg/day - 400 mg/day. Patients then received a stable dosage for a further 16 days. In their study, a secondary outcome included measuring mood using the HADS. The study found no difference in anxiety scores or depression scores between the oral ketamine group and placebo group.^36^ More research is needed to assess the safety and efficacy of oral ketamine in cancer patients for the treatment of depression.

In summary, despite a wealth of evidence demonstrating an antidepressant benefit of ketamine in the general population,^2^ the topic remains insufficiently researched in the oncologic population.^20^ Still, several RCTs have demonstrated a reduction in depressive symptoms for at least three days post IV ketamine administration, with one study demonstrating benefit for one month post administration.^21^, ^22^, ^23^, ^24^, ^25^ More research is needed to fully delineate optimal dosing regimens, differences in effectiveness between esketamine and racemic ketamine, and timing of administration. The efficacy and safety of non-intravenous routes, such as nasal, oral, and topical administration has not been thoroughly assessed in cancer patients. Longitudinal studies are required to evaluate ketamine’s sustained effectiveness throughout cancer’s progression and treatment course.

## Pain management

### Analgesic actions of ketamine

In addition to its antidepressant benefits, the analgesic properties of ketamine can be utilized to treat pain in cancer patients. At high doses, its sedative effects are better suited for anesthesia, but at low doses, ketamine primarily exerts an analgesic effect.^37^ As a phencyclidine derivative, it acts as a non-competitive antagonist of NMDA both centrally and peripherally. NMDA antagonism acts as the primary mechanism for the analgesic properties of ketamine. By inhibiting excitatory neurotransmitter of glutamate in the central nervous system, ketamine disrupts the transmission of pain signals and reduces sensitivity to painful stimuli.^38^, ^39^, ^40^, ^42^ NMDA activation plays a role in central sensitization, a process in which the CNS develops a heightened response to painful stimuli, and ketamine may inhibit this process.^38^, ^40^ NMDA activation also blunts the analgesic properties of opioids, both endogenous and exogenous, and blockade of NMDA by ketamine can potentiate opioid analgesia.^41^, ^42^ In addition to NMDA, ketamine interacts with various other receptors, including nicotinic, muscarinic, opioid, monoaminergic, GABA, adenosine, and AMPA receptors.^37^, ^38^, ^39^, ^40^ The direct effects of these interactions on pain relief remain unclear, and these complex pathways are part of a larger, integrated system of neurotransmission and signaling. Further investigation is required to clearly define the role of ketamine within this system beyond NMDA antagonism.^37^ Research suggests that ketamine may limit the activation of astrocytes and microglia, which can contribute to the suppression of pain transmission.^37^, ^40^ Additionally, ketamine has been shown to enhance the descending inhibitory serotonergic pathway, thereby enhancing endogenous antinociception.^41^ There is a recent shift towards using esketamine instead of racemic formulations, which has demonstrated even stronger analgesic effects with fewer side effects.^39^, ^43^

### Ketamine and cancer pain

Pain is among the most prevalent issues in cancer patients, affecting up to 70 % of patients.^44^ Up to 86 % of patients with advanced cancer experience significant pain.^45^ This pain is linked to a reduced quality of life, decreased emotional well-being, and increased healthcare costs.^46^ Over one-third of patients experience cancer-related pain even after curative treatment, and two-thirds of patients with advanced or metastatic cancer report pain.^47^, ^48^ Opioids remain the primary treatment for cancer pain. Approximately 20 % of cancer patients undergoing opioid titration experience refractory pain and have poor analgesic response, and many patients also experience intolerable side effects.^49^, ^50^ Ketamine is used to treat acute and chronic pain in sub-anesthetic doses, and numerous studies have examined its efficacy in providing pain relief for cancer patients.^51^ Ketamine is also currently used to treat several pain syndromes. However, there is limited data on optimal dosages, treatment durations, and routes of administration to establish standard guidelines for best practices.^52^, ^53^ We conducted a literature search in MEDLINE® (via PubMed®), EMBASE, and Scopus from 1 January 1982–20 October 2023. The search resulted in 1454 unique citations and 21 studies were identified.^54^ Only RCTs that were peer-reviewed, English-language, and with rigorous methodologies were included to ensure high-quality data.

## Acute pain after cancer surgery

### IV ketamine

#### Intra-operative administration

In an RCT of 100 patients with rectal adenocarcinoma, Kock et al. observed that IV ketamine, administered intraoperatively as a bolus of 0.5 mg/kg, followed by a continuous infusion of 0.25 mg/kg per hour during surgery significantly reduced short-term postoperative pain assessed at 72 h postop, and even decreased residual pain up to the sixth postoperative month. No difference was found in the group given 0.25 mg/kg ketamine followed by an infusion of 0.125 mg/kg. No adverse events were reported. They concluded that subanesthetic doses of IV ketamine administered during surgery reduces hyperalgesia and is a beneficial addition to a balanced perioperative analgesic strategy.^55^

In a RCT of 60 patients undergoing radical prostatectomy, Chelly et al. found that IV ketamine can be safely and effectively incorporated into a multimodal pain (MM) regimen. They compared two pain management regimens: an experimental group receiving 10 mg of IV ketamine administered after anesthesia induction, a pre-operative paravertebral block, post-operative celecoxib, and access to morphine through a patient-controlled analgesia (PCA) pump, and a control group receiving only morphine through PCA. They found that the MM group reported significantly lower pain ratings at 24 h when compared to group receiving PCA morphine alone, they also reported lower overall morphine use in the MM group. No serious complications were observed in either group.^56^

In a RCT of 85 patients with colonic adenocarcinoma, Lavand'homme et al. found that the combination of intraoperative IV ketamine (0.5 mg/kg bolus followed by 0.25 mg/kg/h until the end of surgery) and intraoperative epidural provided effective preventive analgesia after major digestive surgery for neoplastic colonic resections, with effects lasting up to one year. All study groups received ketamine, with epidural regimens differing between groups. No adverse events were reported.^57^

In a RCT of 184 patients with breast cancer, Kang et al. found that intraoperative ketamine (0.5 mg/kg bolus followed by 0.12 mg/kg/h until the end of surgery) significantly reduced pain for three months post-surgery compared to the control group the received IV saline. However, the reduction in pain was not considered clinically significant, as there were no differences in quality of life between the groups.^58^ The ketamine group experienced a delayed extubation time, but otherwise no adverse events were reported.

In contrast to the previously mentioned studies, a RCT of 90 patients with breast cancer by Mahran et al. found no difference in post-operative pain 24 h after surgery between two groups of patient’s undergoing breast surgery: one that received 150 mg of oral pregabalin 1 h before surgery and another that received 0.5 mg/kg of IV ketamine with the induction of anesthesia followed by 0.25 mg/kg/h IV ketamine throughout surgery. However, both groups demonstrated reduced morphine consumption compared to the placebo group, indicating that both IV ketamine and oral pregabalin may reduce postoperative opioid consumption in breast cancer surgery.^59^

#### Post-operative administration

In a RCT of 57 patients with bone and soft tissue cancer, Kollender et al. found that using ketamine along with low dose morphine in a PCA pump (5 mg ketamine + 1 mg morphine with 7-minute lockout) significantly reduced post-operative pain for 96 h following orthopedic oncologic surgery when compared to high dose morphine PCA alone (1.5 mg morphine with 7-minute lockout). They concluded that using subanesthetic ketamine with low dose morphine results in lower and more stable pain scores, a reduction in morphine use, and less dependence on postoperative IV-PCA. The ketamine group did not report hallucinations or other adverse events, and they reported significantly less nausea and vomiting rates than the morphine group.^60^ Nesher et al. conducted a similar study in patients 41 undergoing thoracotomy for minimally invasive direct coronary artery bypass or for lung tumor resection. Using the same dosage regimens described by Kollender et al.l, they found that patients receiving a PCA combination of a reduced morphine with subanesthetic ketamine resulted in patients reporting lower pain scores for 72 h after surgery compared to those receiving the standard morphine dose alone. The combination therapy also resulted in fewer adverse side effects and a 45 % reduction in morphine consumption. No adverse events where reported.^61^

### Intrathecal ketamine

Intrathecal ketamine for post-operative pain has also been studied in the oncologic population. Abd El-Rahman et al. examined how intrathecal ketamine (0.1 mg/kg) or morphine (0.3 mg of morphine), and their combination with Bupivacaine affected morphine PCA consumption following major abdominal cancer surgery in 90 patients. Results showed that while all groups experienced effective pain management, combining ketamine and morphine intrathecally led to a significantly greater reduction in the amount of postoperative morphine requested in the 24 h following surgery. The only side effect reported was increased sedation.^62^ In a similar study consisting of 90 patients with abdominal cancer, Mohamed et al. found that combining intrathecal dexmedetomidine and ketamine (0.1 mg/kg) with bupivacaine extended the time to first analgesia request and reduced total morphine consumption for 24 h postop compared to using either drug alone, with only an increase in sedation as a notable side effect.^63^

### Ketamine in nerve blocks

Studies have also assessed incorporating ketamine into nerve blocks. In an RCT, Othman et al. compared the analgesic efficacy and safety of modified pectoral nerve blocks with ketamine (1 mg/kg) plus bupivacaine vs bupivacaine alone in 60 patients undergoing breast cancer surgery. They found that adding ketamine to pectoral blocks prolonged the time until patients first requested analgesia and reduced total opioid consumption for 48 h after surgery without serious side effects. However, there was no observed difference in pain scores in the 48 h post-op period between the two groups.^64^ In a similar RCT of 90 patients undergoing breast cancer surgery, Kamal et al. found that ketamine-bupivacaine blocks lowered acute post-operative pain in a dose-dependent manner (1.0 mg/kg showed greater benefits than 0.5 mg.kg group) and decreased pain scores one month following surgery when compared against bupivacaine only blocks. No adverse events were reported.^65^ Finally, consistent with earlier studies, Shah et al. reported lower pain scores in a cohort of 70 patients receiving pectoralis nerve blocks with ketamine (0.5 mg/kg) during radial mastectomies.^66^

### Intramuscular ketamine

Rakhman et al. examined whether intramuscular ketamine administered pre-operatively could elicit a postop analgesic affect in patients undergoing tumor resections. Their RCT consisted of 120 patients with abdominal tumors, soft tissue sarcomas, or small bone tumors and they split groups into those receiving one, two, or three escalating doses of ketamine before surgery. Doses were administered 17-, 11- and 4-h pre-op in the group receiving three doses, and 11 and 4 h before surgery in the group that received two doses. They found that patients receiving either two (10 mg, 25 mg) or three (5 mg, 10 mg, 25 mg) doses of ketamine reported significantly lower pain scores and reduced morphine use compared to the placebo group. Patients were monitored for 48 h after surgery. Dizziness was a reported side effect in the group that received 25 mg ketamine; otherwise, no other adverse events were reported.^66^

### Local infiltration

Ketamine incorporation in local anesthetic has also been assessed. Abd El-Rahman et al. compared the postoperative effects of local wound instillation of ketamine (1 mg/kg), intramuscular ketamine (1 mg/kg), and placebo in patients undergoing thyroidectomies for thyroid cancer. Group I (local ketamine) showed lower morphine consumption in the 24 h after surgery and a delayed first request for analgesia compared to Group II (intramuscular ketamine) and placebo. Pain ratings were lower in Groups I and II compared to placebo. The findings suggest that local wound ketamine instillation may provide superior postoperative analgesia with fewer side effects than intramuscular ketamine and placebo.^67^

Table 2 summarizes multiple randomized controlled trials (RCTs) that evaluated the use of ketamine for acute pain in cancer patients. Studies thus far have shown that the intravenous route is the most commonly utilized and effective method for treatment of acute pain in surgical cancer patients. Incorporation into nerve blocks and intrathecally has also shown promising results. While, local infiltration and intramuscular methods have yielded mixed results, they may offer patients fewer intimidating alternatives for those who are averse to the intravenous route.

**Table 2:** Summary of randomized controlled trials assessing ketamine for acute pain in cancer patients.

**Authors**	**Number of Patients**	**Type of Cancer**	**Administration Route**	**Dosage**	**Experimental Group(s)**	**Control Group**	**Outcomes Associated with Pain**	**Study Conclusion**
Kock et al. (2001)^55^	100	Rectal adenocarcinoma	IV	0.5 mg/kg bolus, followed by 0.25 mg/kg/h until end of surgery	Intra-op ketamine + morphine PCA	Morphine PCA	Reduced hyperalgesia, PCA requirements, and residual pain	Intra-op IV ketamine reduces wound hyperalgesia, PCA requirements, and residual pain
Chelly et al. (2011)^56^	60	Prostate	IV	10 mg of IV ketamine given after anesthesia induction	MM regimen of intra-op ketamine, a pre-operative paravertebral block, post-op celecoxib and access to morphine through a patient-controlled analgesia (PCA) pump	Morphine PCA	Lower pain ratings at 24 h when compared to group receiving PCA morphine, lower overall morphine use in the MM group	Intra-op IV ketamine can be safely and effectively incorporated into a multimodal pain (MM) regimen
Lavand'homme et al. (2005)^57^	85	Colonic adenocarcinoma	IV	0.5 mg/kg bolus, followed by 0.25 mg/kg/h until end of surgery	All groups received ketamine; epidurals different between groups	N/A	N/A	Combined with IV ketamine, intra-op epidural analgesia provides effective preventive analgesia after major digestive surgery.
Kang et al. (2020)^58^	184	Breast	IV	0.5 mg/kg bolus, followed by 0.12 mg/kg/h until end of surgery	Intra-op ketamine	IV Saline	Reduced pain for three months post-surgery	Intra-op ketamine reduced pain for three months post-surgery to a statistically significant amount, but not clinically significant amount as no differences were found in quality of life between the groups
Mahran et al. (2015)^59^	90	Breast	IV	0.5 mg/kg bolus, followed by 0.25 mg/kg/h until end of surgery	Intra-op ketamine OR Oral pregabalin (150 mg) 1 h before surgery	Placebo capsule before induction and IV saline	Reduced morphine consumption in ketamine and pregabalin groups, no difference in pain in first 24 h	IV ketamine with induction of anesthesia and preoperative oral pregabalin reduces postoperative opioid consumption in breast cancer surgery
Kollender et al. (2008)^60^	57	Bone and soft tissue	IV-PCA	1 mg morphine + 5 mg ketamine/bolus, 7 min lockout time	Morphine (reduced dose) + Ketamine PCA	Morphine (standard dose) PCA	Lower and more stable pain scores, reduced morphine consumption, shorter PCA dependence, better physical performance 96 h post-op	Subanesthetic ketamine with reduced dose morphine PCA results in better pain control, reduced morphine use, shorter PCA dependence, and better early physical performance
Nesher et al. (2009)^61^	41	Lung	IV-PCA	1 mg morphine + 5 mg ketamine/bolus, 7 min lockout time	Morphine (reduced dose) + Ketamine PCA	Morphine (standard dose) PCA	Lower pain scores, reduced morphine use, lower IV-PCA activation rate, fewer adverse effect	Subanesthetic ketamine with reduced morphine PCA provides effective pain control with fewer side effects and a reduction in morphine consumption
Abd El-Rahman et al. (2018)^62^	90	Abdominal	Intrathecal	0.1 mg/kg ketamine (alone) or with 0.3 mg of morphine	Group M: bupivacaine + morphine; Group K: bupivacaine + ketamine; Group M, K: bupivacaine + morphine + ketamine	N/A	Reduced total PCA morphine consumption, prolonged time to first request of analgesia, reduced VAS in Group M + K	Intrathecal ketamine and morphine reduces morphine PCA consumption compared to either drug alone, providing good postoperative analgesia with no side effects apart from sedation
Mohamed et al. (2016)^63^	90	Abdominal	Intrathecal	0.1 mg/kg ketamine or 5 µg dexmedetomidine + 0.1 mg/kg ketamine	Group I: bupivacaine + dexmedetomidine Group II: bupivacaine + ketamine Group III: bupivacaine + dexmedetomidine + ketamine	N. A	Group III experienced a longer duration before requesting their first dose of analgesia, consumed less morphine, and reported lower postoperative pain scores.	The combination of intrathecal dexmedetomidine and ketamine provides superior postoperative analgesia, prolongs time to first request of rescue analgesia, and reduces total PCA morphine consumption
Othman et al. (2016)^64^	60	Breast	Pectoral Nerve block	30 mL of 0.25 % bupivacaine + ketamine hydrochloride (1 mg/kg)	Ketamine + Bupivacaine nerve block	Bupivacaine nerve block	Prolonged time to first request of analgesia and reduced total morphine consumption in the Ketamine group	Addition of ketamine to Pectoral blocks prolongs the time to first request of analgesia and reduces total opioid consumption without serious side effects
Kamal et al. (2019)^65^	90	Breast	Pectoral Nerve block	20 mL bupivacaine 0.25 % + ketamine 0.5 mg/kg OR 20 mL bupivacaine 0.25 % + ketamine 1 mg/kg	Ketamine + Bupivacaine nerve block	Bupivacaine nerve block	Prolonged time to first request of analgesia, decreased total morphine consumption, lower pain scores	Ketamine-bupivacaine in thoracic paravertebral block controls acute postoperative pain in a dose-dependent manner
Rakhman et al. (2011)^66^	120	Unspecified	Intramuscular	K1 group: 25 mg ketamine at 4 h preoperatively K2 group: 10 mg and 25 mg ketamine at 11 and 4 h preoperatively K3 group: 5 mg, 10 mg, and 25 mg ketamine at 17, 11, and 4 h preoperatively	IM Ketamine	IM Saline	K2 and K3 reported lower pain scores, reduced morphine consumption.	Two or three escalating subanesthetic doses of IM ketamine administered consecutively hours before surgery reduced postoperative pain and morphine consumption over 48 h
Abd El-Rahman et al. (2018)^67^	90	Thyroid	Topical	Group I: 1 mg/kg ketamine in 10 mL normal saline instilled in the wound Group II: 1 mg/kg IM ketamine	Topical ketamine, IM ketamine	Saline	Reduced total morphine consumption and delayed first request of analgesia, Decreased pain at rest and with movement. Topical superior to IM	Local wound ketamine instillation provides superior postoperative analgesia with a lower incidence of side effects compared to IM ketamine and placebo following total thyroidectomy.

## Ketamine and chronic pain in cancer patients

While opioids remain the primary therapy for managing cancer pain, many patients develop tolerance to their effects and may benefit from alternative treatments. It is not clear what the possible long-term effects are of chronic pain management with ketamine, and its effectiveness in treating chronic cancer pain remains uncertain. One of the strongest arguments for using ketamine as an adjunct or alternative to opioids is its ability to reduce common side effects associated with opioid use, such as nausea, vomiting, constipation, respiratory depression, and pruritis. This section of the review examines ketamine’s efficacy for managing chronic pain in cancer patients.

### Topical administration to treat chemotherapy-induced peripheral neuropathy

Topical administration of ketamine may be the least intimidating option for patients and has a low side effect burden. Ketamine rapidly transverses epidermal and dermal tissues and binds peripheral receptors with minimal systemic absorption.^68^, ^69^ Barton et al. performed a multi-institution RCT assessing a topical slurry of baclofen, amitriptyline, and ketamine (1.5 %) applied twice per day versus placebo in 203 patients with chemotherapy induced peripheral neuropathy (CINP). Results showed an improvement in both sensory and motor neuropathic symptoms when assessed four weeks after beginning treatment. The most notable improvements were observed in symptoms such as tingling, cramping, shooting/burning pain in the hands, and difficulty holding a pen. There were no significant side effects or evidence of systemic toxicity.^70^ In contrast, a RCT of 462 patients with CINP found no difference in pain symptoms in patients receiving a slurry of ketamine (2 %) plus amitriptyline versus placebo.^71^ Thus far, evidence for topical ketamine to treat chemotherapy induced peripheral neuropathy is mixed, and more studies are expected.

### Oral administration ketamine to treat chronic cancer pain

Oral ketamine is another intriguing modality that may be effective in treating chronic cancer pain. In a RCT of 60 patients with cancer pain (type not specified), Lauretti et al. evaluated the use of oral ketamine as adjunct to oral morphine for cancer pain management. Oral ketamine, dosed at 0.5 mg/kg every 12 h, was found to be an effective coadjutant analgesic. It also reduced the need for higher doses of morphine with fewer side effects compared to other groups (nitroglycerine patch, dipyrone, and placebo). The findings suggest that combining ketamine with opioids can significantly benefit cancer pain management.^72^

In contrast to the previous study, Fallon et al. performed a study of 214 patients primarily with CINP. Results showed no significant difference in benefit between oral ketamine (40 mg/day–400 mg/day) and placebo. The researchers suggest that ketamine is equivalent to placebo for treating CINP, though future research should focus on patients with central sensitization to potentially identify specific subgroups that might benefit from oral ketamine.^36^ Similarly, in a study of 30 patients with chronic cancer pain, Ishizuka et al. found no enhancement of morphine’s analgesic effects when combined with 10 mg of oral esketamine.^73^

### Subcutaneous ketamine to treat chronic pain

Hardy et al. investigated subcutaneous ketamine’s potential to treat chronic cancer pain in a RCT 185 palliative care patients with cancer. Ketamine was dosed at escalating (100 mg, 300 mg, and 500 mg) doses over a five-day period. They found no significant difference in reported pain severity between the groups, with ketamine associated with nearly twice the incidence of adverse events and more severe grades of adverse events compared to placebo. They concluded that subcutaneous ketamine does not provide benefits as an addition to standard opioid treatment.^74^ McCaffrey et al. evaluated the cost-effectiveness of subcutaneous ketamine versus placebo for chronic, uncontrolled cancer pain in patients unresponsive to opioids and co-analgesics. Results showed no significant difference in response rates between groups receiving escalating doses of ketamine and placebo. Ketamine was associated with higher toxicity, worse health-related quality of life, and higher costs. The study concluded that subcutaneous ketamine, in conjunction with opioids and standard adjuvant therapy, is neither effective nor cost-effective for treating refractory pain in advanced cancer patients.^75^

Table 3 summarizes multiple randomized controlled trials (RCTs) that evaluated the use of ketamine for chronic pain in cancer patients. The data for chronic pain treatment varies, as several studies support ketamine for treatment of chronic pain refractory to opioids, while other studies suggest that there is insufficient data recommending ketamine for chronic cancer pain.^76^, ^77^ Regardless, the discussion on the effectiveness of reduction in opioid requirement by ketamine is extensive. Further randomized controlled trials are required to provide clearer data on the topic and to establish standardized guidelines.

**Table 3:** Summary of randomized controlled trials assessing ketamine for chronic pain in cancer patients.

**Authors, year**	**Number of Patients**	**Type of Cancer**	**Administration Route**	**Experimental group**	**Control Group**	**Outcomes Associated with Pain**	**Conclusion**
Barton et al. (2011)^70^	208	Chemotherapy induced peripheral neuropathy	Topical	(1.5 %) ketamine with 10 mg baclofen, 40 mg amitriptyline HCL in a Pluronic lecithin organogel,	Topical placebo	Reduced tingling, cramping, and shooting/burning pain in the hands. Decreased difficulty within holding a pen.	Topical ketamine with baclofen and amitriptyline reduces symptoms of chemotherapy-induced peripheral neuropathy
Gewandter et al. (2014)^71^	462	Chemotherapy induced peripheral neuropathy	Topical	2 % Ketamine plus 4 % amitriptyline cream.	Topical Placebo	No effect in reducing pain, numbness, or tingling in hands or feet over 6 weeks.	Topical ketamine treatment showed no effect in reducing symptoms.
Lauretti et al. (1999)^72^	60	Unspecified	Oral	0.5 mg/kg ketamine at 12 h intervals as adjunct to 80–90 mg oral morphine daily.	Control group of 80–90 mg oral morphine daily with additional 20 mg morphine daily (10 mg at 12-h intervals).	Placebo and ketamine groups had similar pain ratings. Ketamine at low doses effectively decreased the amount of morphine required to relieve pain.	Low-dose ketamine is an effective adjunct to opioids while also demonstrating an opioid-sparing function.
Fallon et al. (2018)^36^	214	Chemotherapy induced peripheral neuropathy	Oral	Ketamine titrated between 40 mg/d with a maximum dosage of 400 mg/d.	Placebo	There was no difference in the pain ratings between the groups.	Ketamine had the same effect as placebo for relief of cancer-related neuropathic pain.
Ishizuka et al. (2007)^73^	30	Unspecified	Oral	10 mg Esketamine every 8 h with 10 mg morphine	10 mg morphine with placebo	There was no difference in the pain ratings between the groups.	Combining morphine with oral esketamine does not decrease oncologic pain
Hardy et al. (2012)^74^	185	Unspecified	Subcutaneous	Ketamine was dosed at escalating (100 mg, 300 mg, and 500 mg) doses over a five-day period	Placebo	There was no significant difference between proportion of positive outcomes. Ketamine use resulted in nearly twice the number of adverse events per day and were more likely to experience a more severe adverse event per day as well.	When used as an adjunct to opioids in the treatment of chronic uncontrolled cancer pain, ketamine did not have any clinical benefit.
McCaffrey et al. (2019)^75^	185	Unspecified	Subcutaneous	Ketamine was dosed at escalating (100 mg, 300 mg, and 500 mg) doses over a five-day period.	Placebo	Ketamine participants had higher rates of toxicity and worse health-related quality of life. Estimated total means of cost were higher for the ketamine group.	Subcutaneous ketamine as an adjunct to opioids was not cost effective and was not effective for relief of chronic uncontrolled cancer-related pain.

### Safety profile and considerations when using ketamine for pain management

Numerous studies have shown that low-dose ketamine, used in the acute treatment of pain, is safe and associated with fewer adverse events compared to both placebo and high-dose ketamine. Side effects can include impairment of perception of reality, hallucinations, derealization, vivid dreams, dissociation, paranoia, panic attacks, and memory deficits. It is important to note, however, that these side effects most commonly occur with higher dosages used for anesthesia and have been proven to have reduced incidence in lower dosages utilized for pain management.^52^, ^76^, ^77^ Evidence shows that patients treated perioperatively with intravenous ketamine results in an overall reduction in opioid usage post-hospital stay for pain management.^76^ Considering this, the potential side effect burden of ketamine must be weighed against potential complications of opioid use, which include nausea, vomiting, constipation, pruritis, urosepsis, and respiratory depression. Ketamine is not recommended for patients who have any signs or symptoms of preexisting psychosis, liver disease, and should be avoided or closely monitored in patients with cardiovascular diseases as the drug elicits a stimulatory effect.^78^ Regardless of preexisting health status, all patients should be closely monitored for any psychological, cardiovascular, hepatic, or renal side effects.^78^

## Conclusion and future directions

Research on the effects of ketamine on depression in cancer patients remains limited. Five randomized controlled trials (RCTs) consistently indicate that administering a single pre-operative or intraoperative dose of ketamine alleviates postoperative depression in cancer patients.^21^, ^22^, ^23^, ^24^, ^25^ However, all these studies have been conducted in China. Given that patients from diverse ethnic backgrounds may exhibit varying rates of depression and potentially different responses to ketamine treatment, there is a need for multicenter or multinational research to rigorously evaluate the safety and efficacy of ketamine in treating depressive symptoms in cancer patients.

Pain research presents significant challenges due to the complex, multidimensional, and subjective nature of pain, which is influenced by differing causes, mental health comorbidities, and variations in treatment approaches.^79^ Pain assessment remains largely subjective, relying on diverse scales that complicate consistent measurements of pain analysis and clinical improvements. These difficulties may contribute to the conflicting findings observed in current research, most notable in non-intravenous ketamine for acute pain relief.

Research on ketamine’s effects in managing chronic cancer pain remains limited and underexplored. Existing studies show variability in methodology for assessing and diagnosing chronic cancer pain. This inconsistency underscores the need for more robust, multicenter trials with standardized, objective measurements to evaluate pain relief and clinical outcomes from ketamine treatment.

In summary, ketamine shows promise in managing both depression and acute pain in cancer patients when administered intravenously at sub-anesthetic doses. However, ketamine’s efficacy in treating chronic cancer pain remains inconsistent. Alternate administration methods, such as intranasal and oral routes, are insufficiently studied for depression management, while local infiltration and intramuscular routes have produced mixed results for acute pain relief. Further research is needed to fully explore their potential. Overall, ketamine and esketamine offer a promising option for addressing the complex needs of cancer patients.

## CRediT authorship contribution statement

**Sierra Barber:** Writing – original draft. **Heather Jim:** Writing – review & editing, Writing – original draft. **Jeffery Huang:** Writing – review & editing, Writing – original draft, Supervision, Conceptualization. **Michael Bodnar:** Writing – review & editing, Writing – original draft, Investigation, Conceptualization. All authors have read and agreed to the published version of the manuscript.

## Disclosure statement

Not applicable.

## Ethical statement

Not applicable.

## Funding

This work was supported by the National Institute of Health (Grant number R01CA219389).

## Declaration of competing interest

Jeffrey Huang has the position of Associated Editor for JATM, and was blinded from reviewing or making decision for the manuscript. Heather Jim has provided consulting services for SBR Bioscience.

## Data Availability

Not applicable.
